# Pd on poly(1-vinylimidazole) decorated magnetic S-doped grafitic carbon nitride: an efficient catalyst for catalytic reduction of organic dyes

**DOI:** 10.1038/s41598-020-70457-5

**Published:** 2020-08-10

**Authors:** Masoumeh Dorraj, Samahe Sadjadi, Majid M. Heravi

**Affiliations:** 1 Gas Conversion Department, Faculty of Petrochemicals, Iran Polymer and Petrochemicals Institute, PO Box 14975-112, Tehran, Iran; 2 Department of Chemistry, School of Science, Alzahra University, Vanak, PO Box 1993891176, Tehran, Iran

**Keywords:** Chemistry, Catalysis, Heterogeneous catalysis

## Abstract

A novel magnetic catalyst, (SGCN/Fe_3_O_4_/PVIs/Pd) was synthesized by growing of poly(1-vinylimidazole) on the surface of ionic liquid decorated magnetic S-doped graphitic carbon nitride, followed by stabilization of palladium nanoparticles. Catalytic activity of the prepared heterogeneous catalyst was explored for the catalytic reduction of hazardous dyes, methyl orange and Rhodamine B, in the presence of NaBH_4_. Besides, the effects of the reaction variables on the catalytic activity were investigated in detail. The kinetics study established that dye reduction was the first order reaction and the apparent activation energy was calculated to be 72.63 kJ/mol and 68.35 kJ/mol^1^ for methyl orange and Rhodamine B dyes, respectively. Moreover, ΔS^#^ and ΔH^#^ values for methyl orange were found to be − 33.67 J/mol K and 68.39 kJ/mol respectively. These values for Rhodamine B were − 45.62 J/mol K and 65.92 kJ/mol. The recycling test verified that the catalyst possessed good stability and reusability, thereby making it a good candidate for the catalytic purposes. Furthermore, a possible catalytic mechanism for dye catalytic reduction over SGCN/Fe_3_O_4_/PVIs/Pd was proposed.

## Introduction

Dyes and pigments are widely used in cosmetics, textiles, paper, printing and many other industries. It is thus not surprising that organic dyes such as methyl orange (MO) and Rhodamine B(RhB) are one of the most common anthropogenic water pollutants in industrial effluent. Most of these compounds are acutely toxic, mutagenic and either known or suspected carcinogens^1, 2^. Many technologies have been designed for the treatment of wastewater containing organic dyes, including adsorption^3^, coagulation^3, 4^ and reverse osmosis^5^. However, the mentioned methods are not sufficient and effective for dye catalytic reduction to nonhazardous products^6^. As a consequence, searching for an effective and suitable approach for the efficient removal of dyes is extremely essential. Chemical reduction method in the presence of metal nanoparticles and NaBH_4_ is considered to be a feasible and potential approach for dye decolonization due to its advantages of low-cost, high-efficiency, and easy operation^7, 8^.

Noble metal based nanomaterials have recently gained significant attention, since they have outstanding physicochemical properties and great potentials in various fields such as optical, catalytic, biomedical and environmental application^9–12^. Among them, palladium nanoparticles (Pd NPs) are the most promising nanoparticles served in several industries and academic synthetic chemistry laboratories as effective catalysts for many organic reactions^13^. However, in the practical application, noble metal NPs can agglomerate easily because of their large surface area, which subsequently results in poor catalytic activity and durability^13–15^.

To solve this problem, a large number of supporting materials, such as polymers^12, 16^, metal oxides^17–20^, clays^21^ and carbon based materials^22–24^ have been used to support and stabilize NPs. Among many types of carbon based support, graphitic carbon nitride (g-C_3_N_4_) has been considered as an ideal support for various metal nanoparticles^25–29^. However, the pristine g-C_3_N_4_, still suffers from unsatisfactory adsorption performance owing to its insufficient active sites and limited specific surface area. An important strategy for improving the adsorption capacity of g-C_3_N_4_ is enhancing the active sites by doping heteroatoms (e.g. S, O, B, P)^30–33^. In addition, the element doping approach can increase some defects of bulk g-C_3_N_4_^34, 35^, thus providing more active sites for binding target ions. Among doped g-C_3_N_4_, S-g-C_3_N_4_ (SGSN) showed improved electron transfer and catalytic performance^36, 37^. For example, Li et al.^33^ showed that doping g-C_3_N_4_ with S can facilitate the adsorption ability of Pb(II) since soft S ligands serve as Pb(II) scavenger. Thus, use of SGCN can improve the performance of graphitic carbon as a support.

Magnetic nanoparticles offer significant promise due to their magnetic properties, allowing for easy and fast recovery with a conventional magnet^38, 39^. Therefore, the design and development of new magnetic catalysts that can be easily separated from the solution is of great importance.

One of the major problems associated with the immobilized metallic heterogeneous catalysts is the low catalyst loading and high catalyst leaching. In the conventional immobilization of metal NPs on a solid support, only one layer of the support surface is available and consequently the metal loading is expected to be low. This problem can be addressed by coating of solid surfaces by functional polymers^17, 40, 41^. Among various functional polymers, poly (1-vinylimidazole) (PVI) has been intensively studied as a compound for anchoring metal ions in solution. In some cases, PVI has been employed due to its complex formation capability^42^, whereas other studies focused on the utility of PVI for the preparation of polymer-grafted nanoparticles^41^. Vinylimidazole (VI) has also been successfully used for the synthesis an ion-imprinted silica supported organic–inorganic hybrid for heavy metal ions removal^43^ and carrying metal-chelated beads for reversible use in yeast invertase adsorption^44^.

Ionic liquids, ILs, are a class of very applicable organic salts that can be applied as catalysts, carbon precursor and solvents^45–47^. These organic salts can also be successfully used for the immobilization of catalytic species on the supports^48^.

**Figure 1:**
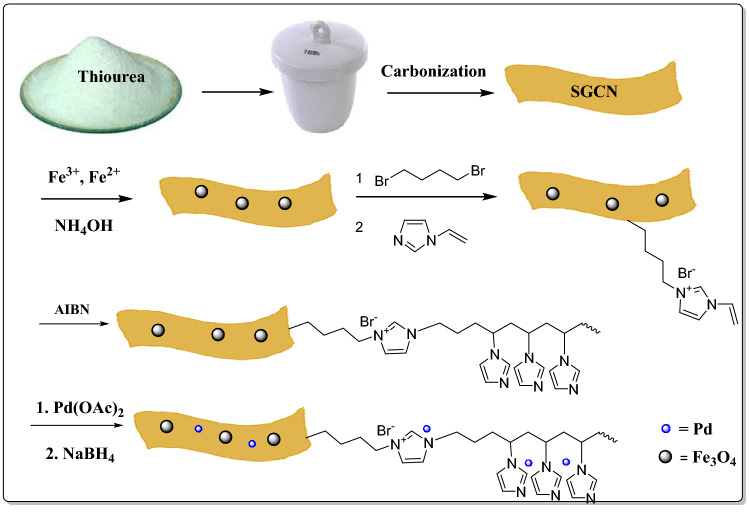
The schematic procedure of the synthesis of the SGCN/Fe_3_O_4_/PVIs/Pd catalyst.

In the pursuit of our research on the design of novel hybrid catalytic systems based on g-C_3_N_4_^49–51^ and IL^52–54^, herein, we report the synthesis of a novel magnetic heterogeneous hybrid catalyst. In this catalytic system, magnetic SGSN was functionalized with vinyl IL and then polymerized with vinyl imidazole to form PVI. The resulting hybrid was then applied as a support for Pd immobilization, (Fig. 1). The prepared SGCN/Fe_3_O_4_/PVIs/Pd nanocomposite was then used as a magnetic catalyst for the catalytic reduction of MO and RhB in the presence of NaBH_4_. In addition, the kinetic and the effects of the reaction temperature, the catalyst amount and the reaction time on the removal of MO and RhB were investigated. Moreover, the recyclability of SGCN/Fe_3_O_4_/PVIs/Pd was studied.

## Result and discussion

### Catalyst characterizations

**Figure 2:**
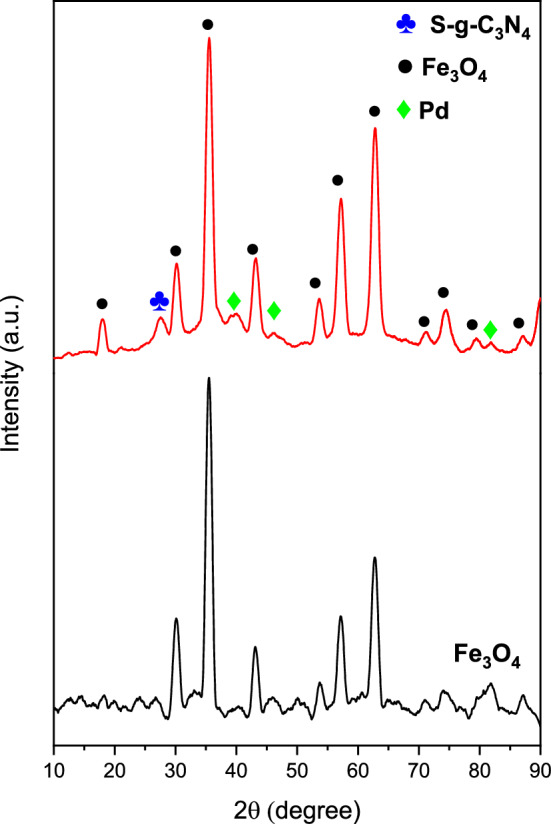
XRD pattern of SGCN/Fe_3_O_4_/PVIs/Pd nanocomposite.

The X-ray diffraction (XRD) was applied to monitor the crystal phase of SGCN (Fig. S1) and SGCN/Fe_3_O_4_/PVIs/Pd (Fig. 2). Typically, the strongest peak observed for SGCN at 2θ = 27.6° can be representative of interlayer stacking of aromatic system (002). A small diffraction peak at 2θ =  ~ 13.1° can be indexed to the (100) plane and assigned to the in-plane aromatic structural packing^33, 55^. Regarding SGCN/Fe_3_O_4_/PVIs/Pd nanocomposite XRD pattern, the peak at 2θ = 27.6° had a considerably reduced intensity and became broader, while the peak at 13.1° vanished, owing to the introduction of Fe_3_O_4_, Pd NPs or the interaction of the Pd NPs and SGCN in SGCN/Fe_3_O_4_/PVIs/Pd nanocomposite^33, 38^. Eleven characteristic diffraction peaks of Fe_3_O_4_ are found in XRD pattern of SGCN/Fe_3_O_4_/PVIs/Pd nanocomposite (denoted as black circles)^56^, suggesting that Fe_3_O_4_ has been successfully immobilized on S-g-C_3_N_4_. The indexed (111) and (200) diffraction peaks at 39.66°, 46.46° and 82.08° are assigned to the Pd NPs (JCPDS No. 46–1,043), corresponding to the face centered cubic (fcc) Pd lattices^57^.

**Figure 3:**
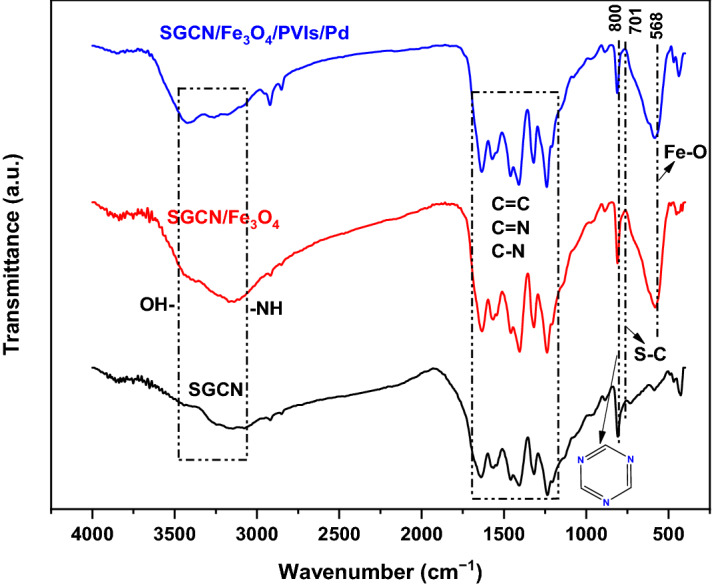
FT-IR spectra of SGCN, SGCN/Fe_3_O_4_ and SGCN/Fe_3_O_4_/PVIs/Pd nanocomposite.

The FTIR spectra of SGCN, SGCN/Fe_3_O_4_ and SGCN/Fe_3_O_4_/PVIs/Pd nanocomposite are presented in Fig. 3. FTIR spectra of all the above mentioned materials presented similar absorption bands at 800 and 1,200–1,600 cm^−1^, which are attributed to triazine units, aromatic –C=C/–C=N/–C–N bonds, as well as the band at 3,100–3,500 cm^−1^ that can be assigned to –NH and –OH groups^30, 33^. The presence of S–C bond at 701 cm^-1^ in the FTIR spectrum of SGCN, implied the successful incorporation of sulfur into g-C_3_N_4_ structure^33, 37^. As for SGCN/Fe_3_O_4_ and SGCN/Fe_3_O_4_/PVIs/Pd, the absorption band at 568 cm^−1^ can be due to Fe–O bond^38^.

**Figure 4:**
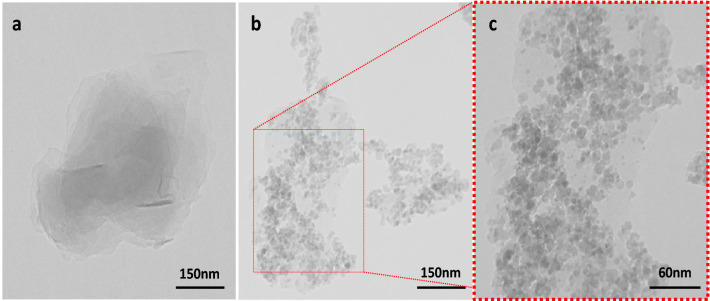
TEM images of (**a**) SGCN and (**b**, **c**) SGCN/Fe_3_O_4_/PVIs/Pd nanocomposite.

The morphologies of SGCN and SGCN/Fe_3_O_4_/PVIs/Pd samples were examined with TEM as shown in Fig. 4a–c. Figure 4a shows the film-like morphology with a layered structure of the SGCN (a unique folded graphene like structure composed of spatially interconnected nanosheets). Figure 4b,c corroborated that Fe_3_O_4_ and Pd nanoparticles are highly dispersed on the surface of the support. Furthermore, the EDS analysis of SGCN/Fe_3_O_4_/PVIs/Pd nanocomposite (Fig. S2) showed the presence of Fe and Pd atoms, affirming successful incorporation of metallic nanoparticles on the hybrid support. Moreover, the presence of S, C and N is indicative of SGCN. Notably, the absence of Br atom can be ascribed to its low content.

FESM image and elemental mapping analysis of SGCN/Fe_3_O_4_/PVIs/Pd nanocomposite were also recorded, Fig. S3. It was found that both Pd and magnetic nanoparticles were dispersed homogeneously on the composite.

Magnetic properties of the Fe_3_O_4_ and SGCN/Fe_3_O_4_/PVIs/Pd samples were investigated at room temperature, Fig. S4. It was confirmed that the magnetization saturation of SGCN/Fe_3_O_4_/PVIs/Pd was 38.42 emu/g, lower than that of Fe_3_O_4_ (51.3 emu/g). This result can be justified by considering the fact that Fe_3_O_4_ nanoparticles were embedded in the support that is a non-magnetic compound^58^.

Fig. S5 showed the thermogravimetric analysis (TGA) results of SGCN and SGCN/Fe_3_O_4_/PVIs/Pd. As for SGCN, upon increase of temperature up to 550 °C, the sublimation or decomposition of SGCN initiated. This process is completed at 650 °C. For SGCN/Fe_3_O_4_/PVIs/Pd nanocomposite, however, the stability of the nanocomposites greatly decreased (the decomposition temperature is shifted to 449.1 °C, which is lower than that of SGCN). This is assigned to the oxidation and decomposition of PVIs.

### Kinetic and thermodynamic studies of the reduction reaction of dyes in the presence of SGCN/Fe_3_O_4_/PVIs/Pd catalyst

The catalytic activity of the SGCN/Fe_3_O_4_/PVIs/Pd nanocomposite was evaluated in the reduction reaction of MO and RhB dyes with NaBH_4_ as the reducing agent and the progress of reaction monitored with the help of ultraviolet–visible (UV–Vis) absorption spectroscopy. The initial experiments established that in the absence of the catalyst, no reaction progress was apperceived, indicating that the catalyst play an important role in the reduction process. In the next step, the influence of SGCN/Fe_3_O_4_/PVIs/Pd loading on MO and RhB catalytic reduction was assessed. In this regard, the catalytic performances of different amounts of catalyst (1, 2, 3, 4 and 5 mg) were evaluated under similar operating condition (performing the reaction at room temperature, in water as solvent). Experimental results affirmed that the conversion of the reactions increased by the increment of the content of SGCN/Fe_3_O_4_/PVIs/Pd up to an optimum level (2 mg for MO and 4 mg for RhB) and further increase of SGCN/Fe_3_O_4_/PVIs/Pd loading had no remarkable effect on the reaction conversion, Table S1.

**Figure 5:**
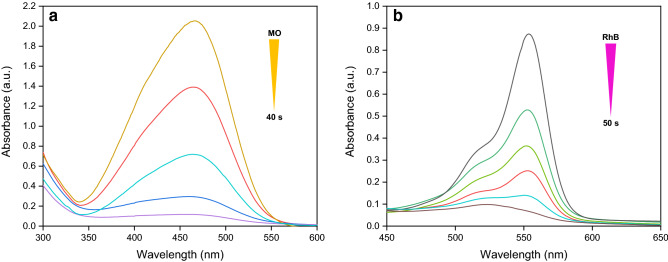
Time-dependent UV–visible spectra for the catalytic reduction of (**a**) MO and (**b**) RhB dyes by NaBH_4_ in the presence of optimum amount of SGCN/Fe_3_O_4_/PVIs/Pd.

The reduction progress for both dyes over time was monitored by measuring the temporal evolution of UV–Vis absorption spectra of the reaction mixtures under SGCN/Fe_3_O_4_/PVIs/Pd catalysis (Fig. 5). As shown, the absorption peaks of the dyes (λ_max_ = 465 nm for MO and λ_max_ = 550 nm for RhB) decreased gradually as the reaction elapsed. This implied high efficiency of SGCN/Fe_3_O_4_/PVIs/Pd for dye decolorization in a short time of the reaction (40 s for MO and 50 s for RhB).

The MO and RhB catalytic reduction processes followed the pseudo-first-order kinetic, which can be described by the following equation^59, 60^:

1 $$\ln {\raise0.7ex\hbox{${C_{0} }$} \!\mathord{\left/ {\vphantom {{C_{0} } C}}\right.\kern-\nulldelimiterspace} \!\lower0.7ex\hbox{$C$}} = kt$$

In that equation, the values of *C*_0_ (dye concentration at the start of the reaction) and *C* (dye concentration at time *t*) can be obtained from the absorbance at t = 0 and *t* (A_0_ and A_t_) respectively. Hence, the values of the rate constant (*k*) for the reduction of dyes can be calculated from the slope of ln (*C*_0_/*C*) vs. time (Fig. S6).

The *k* values for the reduction of both MO and RhB at four different reaction temperatures (293, 298, 303 and 308 K) were similarly measured, reported in Table 1. As tabulated, *k* value of the reaction increased with the increment of the reaction temperature (Table 1). *k* values at different temperatures can be helpful for estimating the activation energies (*E*_*a*_). More exactly, having the Arrhenius equation in hand, Eq. (2), and R and *k* values, *E*_*a*_ can be measured from the plot of ln *k* vs. 1/T as shown in Fig. S7 and Table 1.

2 $$\ln k = \ln A - \left( {{\raise0.7ex\hbox{${E_{a} }$} \!\mathord{\left/ {\vphantom {{E_{a} } {RT}}}\right.\kern-\nulldelimiterspace} \!\lower0.7ex\hbox{${RT}$}}} \right)$$

**Table 1:** The values of thermodynamic and kinetic parameters of reduction reaction of MO and RhB dyes in the presence of the SGCN/Fe_3_O_4_/PVIs/Pd catalyst.

Dye	T (K)	*k* (min^−1^)	E_a_ (kJ/mol)	∆S^#^ (J/mol K)	∆H^#^ (kJ/mol)
MO	293 298 303 308	0.036 0.052 0.115 0.141	72.63	33.67	68.39
RhB	293 298 303 308	0.040 0.080 0.124 0.154	68.35	45.62	5.92

For the calculation of activation thermodynamics parameters, i.e. activation entropy (ΔS^#^) and the activation enthalpy (ΔH^#^), Eyring equation (Eq. 3) and Eyring plot (ln (*k*/T) vs. 1/T), Fig. S8, were exploited.

3 $$\ln \left( {{\raise0.7ex\hbox{$k$} \!\mathord{\left/ {\vphantom {k T}}\right.\kern-\nulldelimiterspace} \!\lower0.7ex\hbox{$T$}}} \right) = \ln \left( {{\raise0.7ex\hbox{${k_{B} }$} \!\mathord{\left/ {\vphantom {{k_{B} } h}}\right.\kern-\nulldelimiterspace} \!\lower0.7ex\hbox{$h$}}} \right) + {\raise0.7ex\hbox{${\Delta S^{\# } }$} \!\mathord{\left/ {\vphantom {{\Delta S^{\# } } R}}\right.\kern-\nulldelimiterspace} \!\lower0.7ex\hbox{$R$}} - {\raise0.7ex\hbox{${\Delta H^{\# } }$} \!\mathord{\left/ {\vphantom {{\Delta H^{\# } } R}}\right.\kern-\nulldelimiterspace} \!\lower0.7ex\hbox{$R$}}\left( {{\raise0.7ex\hbox{$1$} \!\mathord{\left/ {\vphantom {1 T}}\right.\kern-\nulldelimiterspace} \!\lower0.7ex\hbox{$T$}}} \right)$$

In Eq. (3), *k*_*B*_ and *h* are constant and known values. Moreover, the study of the kinetic parameters provided the value of ln (*k*/T). Hence, ΔS^#^ and ΔH^#^ can be evaluated from the intercept and slop of Eyring plot respectively. The measured ΔS^#^ values for the reduction reactions of MO and RhB were assessed as -33.678 and −45.626 J/mol K, respectively. ΔH^#^ values of the reduction reactions were measured as 68.397 and 65.929 kJ/mol for MO and RhB dyes, respectively (Table 1).

### Mechanism

**Figure 6:**
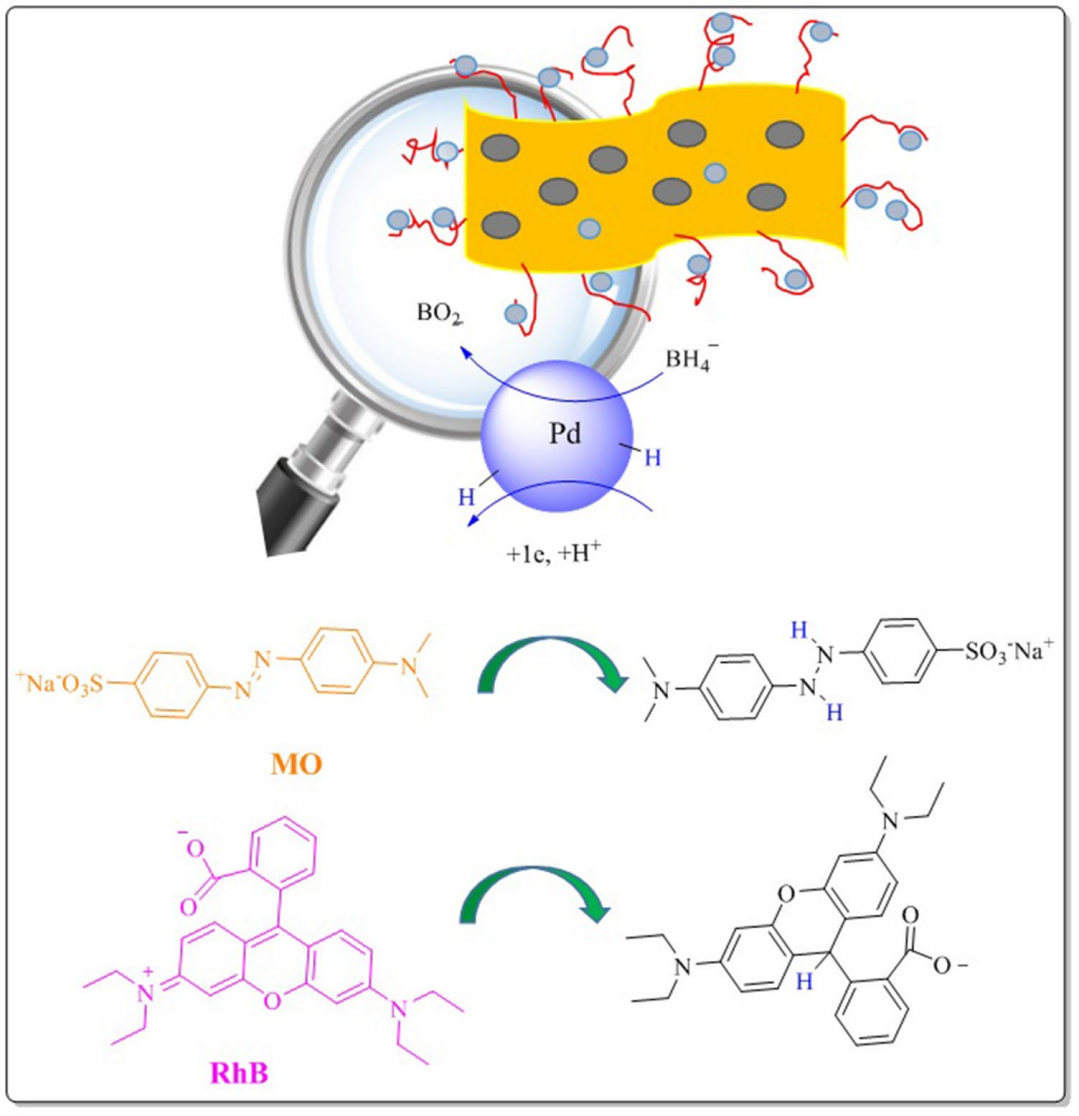
The plausible mechanism for dye reduction.

According to the literature^61^, the plausible mechanism for the reduction of MO and RhB in the presence of SGCN/Fe_3_O_4_/PVIs/Pd can be defined as follow: First, borohydride ions are generated through dissociation of sodium borohydride. Secondly, the as-generated BH_4_^−^ ions are adsorbed on Pd nanoparticles that are the main catalytic species for the reduction reaction. On the other hand, the organic dyes that possess aromatic moieties in their structures can be adsorbed onto SGCN/Fe_3_O_4_/PVIs/Pd through π-π stacking interactions. Thirdly, the adsorbed dyes were reduced by the generated hydride ions, Fig. 6. Finally, the reduced dye will be released from SGCN/Fe_3_O_4_/PVIs/Pd.

### Recyclability

**Figure 7:**
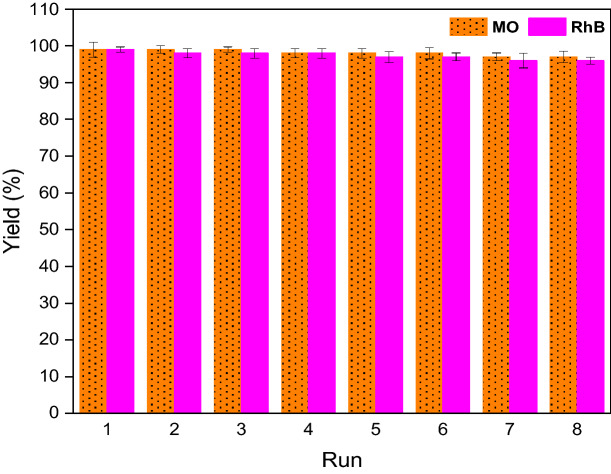
The results of the recyclability of the catalyst for reduction of MO and RhB.

Considering the importance of the reuse of the heterogeneous catalysts in the practical application, the recyclability of SGCN/Fe_3_O_4_/PVIs/Pd for the reduction reaction of both dyes was examined. To accomplish this purpose, SGCN/Fe_3_O_4_/PVIs/Pd was separated by applying an external magnetic from the reaction mixture and then employed for the next reaction run under the same reaction condition. This cycle was repeated up to eight consecutive reaction runs and the obtained yields of each runs for both dyes were measured and compared (Fig. 7). As shown in Fig. 7, SGCN/Fe_3_O_4_/PVIs/Pd could be recycled for 8 reaction runs with only slight loss of the catalytic activity. Furthermore, the Pd leaching of SGCN/Fe_3_O_4_/PVIs/Pd was also investigated for the catalyst reused after eight runs. It was gratifyingly found out that Pd leaching was insignificant (0.01 wt% of initial Pd loading), showing the efficiency and stability of SGCN/Fe_3_O_4_/PVIs for Pd anchoring.

Next, the stability of the recycled SGCN/Fe_3_O_4_/PVIs/Pd was evaluated by recording FTIR spectrum of the recycled SGCN/Fe_3_O_4_/PVIs/Pd after eight runs for the reduction of MO and RhB (Fig. S9a). It was found that the spectra of the recycled SGCN/Fe_3_O_4_/PVIs/Pd for both reactions are similar to that of fresh SGCN/Fe_3_O_4_/PVIs/Pd and no absorbance band has been disappeared upon recycling. Moreover, the TEM analysis of the recycled catalyst after eight cycles did not show major morphological changes (Fig. S9b). These implied that the structure and morphology of SGCN/Fe_3_O_4_/PVIs/Pd were not destroyed after recycling.

## Experimental

The detail of used materials and apparatus is elaborated in SI. Herein, the syntheses of the catalyst and dye reduction are explained.

### Synthesis of the catalyst

#### Synthesis of S-g-C_3_N_4_ nanosheet (SGCN)

Sulphur-doped graphitic carbon nitride (S-g-C_3_N_4_) nanosheets were synthesized by carbonization of thiourea in a muffled furnace^30^. In brief, 10 g of thiourea was placed in crucibles with a cover and calcined at 530 °C in a muffle furnace for 2 h. After calcination, the obtained yellow powder marked as SGCN was ground into fine powder and collected for further usage.

#### Synthesis of the SGCN/Fe_3_O_4_

SGCN/Fe_3_O_4_ nanocomposites was synthesized by precipitation method^6^. Briefly, SGCN powder (0.5 g) was added to 120 mL of distilled water and ultrasonicated (60 W) for 20 min at room temperature. Then, FeCl_3_.6H_2_O (1.37 g) and FeCl_2_·4H_2_O (0.5 g) were dissolved into 12 mL distilled water and the resultant solutions were added to the SGCN suspension under stirring. Stirring was continued for 60 min at 80 °C, and then 10 mL of ammonia solution was added and the mixture was continuously stirred for 60 min, after which the suspension was allowed to cool naturally. Then, the as prepared precipitate was collected by an external magnet, washed with water and ethanol, and dried at 50 °C overnight. The obtained product (named as SGCN/Fe_3_O_4_) was used in the next step.

#### Synthesis of SGCN/Fe_3_O_4_/PVIs Hybrids

SGCN/Fe_3_O_4_ (1.0 g) was dispersed in 45 mL toluene in a clean round-bottom flask. Subsequently, 1,4-dibromobutane (1 mL) was added to the stirring mixture. The resulting mixture was heated, refluxed and kept at 110 °C for 24 h under vigorous agitation. At the end of the reaction, the product was subjected to magnetic separation and washed sequentially with EtOH to thoroughly remove unreacted 1,4-dibromobutane from the surface of SGCN/Fe_3_O_4_. The final product, denoted as SGCN/Fe_3_O_4_ -(CH_2_)_4_Br, was then dried under vacuum at 60 °C overnight to remove the residual solvent. To introduce IL, a mixture containing SGCN/Fe_3_O_4_-(CH_2_)_4_Br (1.0 g) and 1-vinylimidazole (1.5 mL) in 35.0 mL EtOH was stirred for 15 min and then refluxed at 60 °C for 3 h under vigorous agitation to form SGCN/Fe_3_O_4_-(CH_2_)_4_IL. Growth of poly (1-vinylimidazole) (PVI) was achieved via free radical polymerization of 1-vinylimidazole and SGCN/Fe_3_O_4_-(CH_2_)_4_IL in the presence of AIBN (50 mg) as initiator. Polymerization was continued at 80 °C for 24 h under Ar atmosphere. Upon completion of the polymerization reaction, the product was separated magnetically and then washed with ethanol several times. The produced SGCN/Fe_3_O_4_/ PVIs was dried under vacuum at 60 °C to remove the residual solvent.

#### Preparations of SGCN/Fe_3_O_4_/PVIs/Pd

Stabilization of Pd nanoparticles was realized through wet-impregnation procedure^15^. 1.2 g of the SGCN/Fe_3_O_4_/PVIs was agitated in 50 mL toluene. Afterwards, a solution of 0.1 mmol of Pd(OAc)_2_ in 20 mL of toluene was added gradually. After agitation at room temperature for 2 h, a solution sodium borohydride in H_2_O (10 mL, 0.2 N), was added to provide Pd(0) nanoparticles. At the end, SGCN/Fe_3_O_4_/PVIs/Pd was collected, washed with MeOH /EtOH, and dried under vacuum for 13 h. Figure 1 present a schematic illustration of preparation of SGCN/Fe_3_O_4_/PVIs/Pd. Using ICP method, Pd loading was measured as 0.07 mmolg^-1^.

#### Catalytic reduction of dye

To decolorize the dyes, MO or RhB (2 mL), scan content of SGCN/Fe_3_O_4_/PVIs/Pd and sodium borohydride (2 mL, 0.01 M) were mixed in water and stirred. The progress of de-colorization was traced by using time-dependent UV–vis spectroscopy^61^. At the end of the reaction, SGCN/Fe_3_O_4_/PVIs/Pd was collected, rinsed repeatedly with EtOH: H_2_O (1:1) and dried. This experiment was repeated at four temperatures (20, 25, 30 and 35 °C).

## Conclusion

In summary, growth of PVI on IL decorated magnetic SGCN has been reported to furnish an efficient support for stabilization of Pd NPs. The resulting catalyst, SGCN/Fe_3_O_4_/PVIs/Pd, was characterized and applied for the catalytic reduction of MO and RhB in aqueous media at room temperature. The results confirmed high efficiency of the catalyst for reduction of both dyes in almost 1 min, probably because of the chelation properties. The study of the reaction temperature confirmed that the higher the reaction temperature, the faster the reaction proceeded. Moreover, the effect of the catalyst loading was studied to find out the optimum catalyst loading for both reactions. The rate constants of both reactions were calculated at four different temperatures and using some conventional calculation, E_a_, ΔS^#^ and ΔH^#^ values for MO were found to be 68.35 kJ/mol, − 33.67 J/mol K and 68.39 kJ/mol respectively. These values for RhB were 72.63 kJ/mol, − 45.62 J/mol K and 65.92 kJ/mol. Moreover, the recycling of SGCN/Fe_3_O_4_/PVIs/Pd confirmed facile recovery of the catalyst and its excellent recyclability up to eight runs. This catalyst has good potential to real-life applications because of easy handling and separation, and long-term stability. In fact, in this study the chemistry of graphitic carbon nitride was modified by incorporation of heteroatom and introduction of PVI to furnish a potential support for Pd immobilization and developing a catalyst for removal of dyes.

## Supplementary information

Supplementary information.
