# Supplementary material for: Pd on poly(1-vinylimidazole) decorated magnetic S-doped grafitic carbon nitride: an efficient catalyst for catalytic reduction of organic dyes
Source: Sci Rep. 2020 Aug 10;10:13440. doi: 10.1038/s41598-020-70457-5 (PMC7417994; doi:10.1038/s41598-020-70457-5)
Supplement: Supplementary file 1 — Supplementary information. [file 41598_2020_70457_MOESM1_ESM.docx]

**Supporting information**

**Pd on poly(1-vinylimidazole) decorated magnetic S-doped grafitic carbon nitride: An** **efficient catalyst for catalytic reduction of organic dyes**

*Masoumeh Dorraj^1^,* *Samahe Sadjadi^*1^, Majid M Heravi^2^*

**Materials and Instrumentation**

All the used chemicals in this study were of analytical grade and used without further purification. Distilled water was used for the preparation of all solutions. Thiourea (CH_4_N_2_S), FeCl_3_·6H_2_O, FeCl_2_·4H_2_O, 1,4-dibromobutane, 1-vinylimidazole (VI), 2,2’-azobisisobutyronitrile (AIBN), palladium (II) chloride , sodium borohydride (NaBH_4_), methanol (MeOH), ethanol (EtOH), toluene , MO and RhB were purchased from Sigma-Aldrich.

The synthesized hybrid catalyst was characterized using X-ray diffraction (XRD), Fourier transform infrared (FT-IR) spectroscopy, thermogravimetric analysis (TGA), transmission electron microscopy (TEM), energy dispersive X-ray spectroscopy (EDS), VSM (vibrating sample magnetometer) and ICP. XRD pattern of the as-synthesized sample was recorded from 2θ = 8 to 90° on a Siemens (model D5000, Karlsruhe, Germany), using Cu Kα radiation. TGA was performed with a Mettler-Toledo instrument (Leicester, UK) at a heating rate of 10 °C min^-1^ from room temperature up to 800 °C under dynamic nitrogen. FT-IR spectroscopy was undertaken using PerkinElmer Spectrum 65 instrument. TEM images were acquired on a Philips CM30 at 300 kV. To perform this analysis, the samples were prepared in EtOH and allowed to dry in air on a carbon-coated cupper TEM grids. EDS analysis was done using a Bruker XFlash 6 detector. Magnetization measurement was run on a PAR Model 155 vibrating sample magnetometer. To perform ICP analysis and measuring the loading of Pd, ICP-AES Varian, Vista-pro (Salt lake city, Australia) was used.

**Figure S1.** XRD patterns of SGCN.


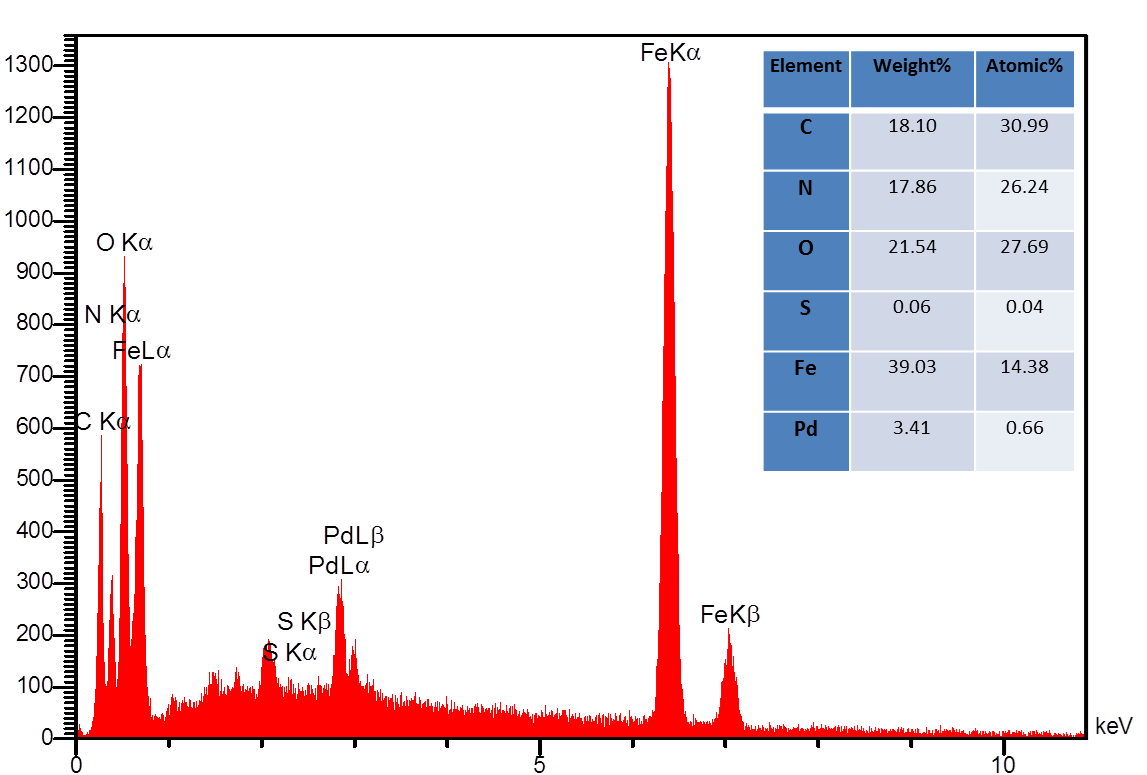


**Figure S2.** EDS analysis of SGCN/Fe_3_O_4_/PVIs/Pd nanocomposite and the quantitative elemental composition of the nanocomposite (the inset).


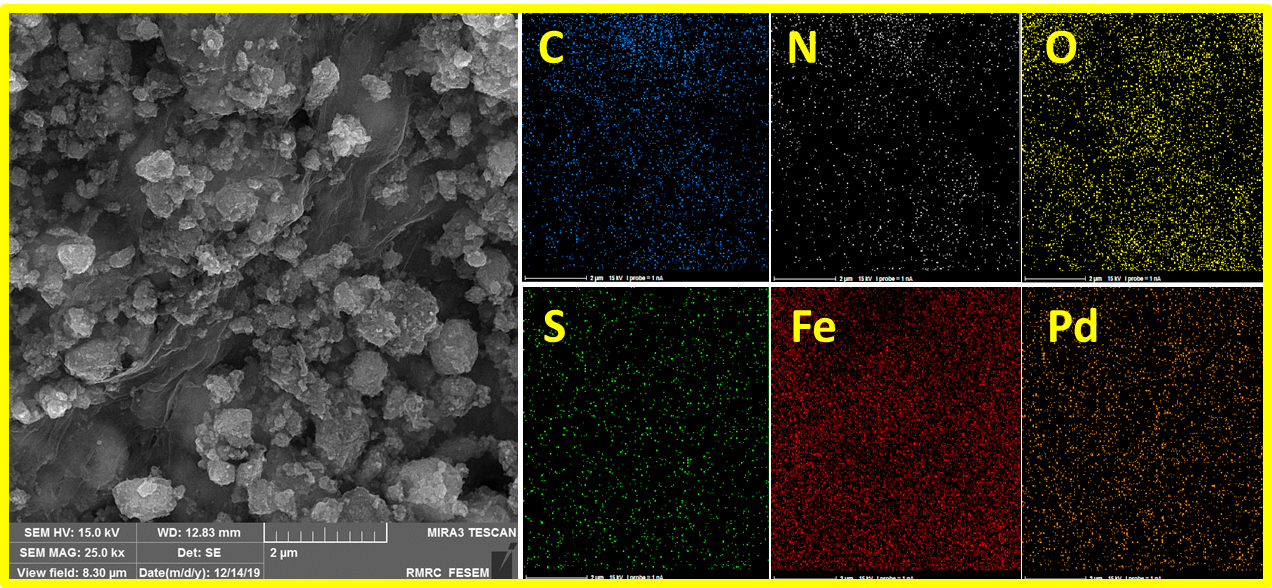


**Figure S3.** FESEM mapping analysis of SGCN/Fe_3_O_4_/PVIs/Pd nanocomposite and the corresponding elements.

**Figure S4.** Magnetization curves of pristine Fe_3_O_4_ and SGCN/Fe_3_O_4_/PVIs/Pd.

**Figure S5.** The termogravimetric curves of SGCN and SGCN/Fe_3_O_4_/PVIs/Pd.

**Table S1.** The optimization of the amount of SGCN/Fe_3_O_4_/PVIs/Pd for reduction of MO and RhB

| Dye | Catalyst amount (mg) | Conversion (%) |
| --- | --- | --- |
| MO | 1 | 65 |
|  | 2 | 99 |
|  | 3 | 99 |
|  | 1 | 52 |
|  | 2 | 64 |
| RhB | 3 | 81 |
|  | 4 | 99 |
|  | 5 | 99 |

**Figure S6.** The diagrams of ln (C_0_/C) vs. time (s) for reduction of (a) MO and (b) RhB dyes at four temperatures.

**Figure S7.** The diagrams of ln *k* vs. 1/T for reduction of (a) MO and (b) RhB dyes at four temperatures.

**Figure S8.** The diagrams of ln *k*/T vs. 1/T for reduction of MO (a) and RhB (b) dyes at four temperatures.

**Figure S9.** (a) The FTIR spectra of SGCN/Fe_3_O_4_/PVIs/Pd fresh (black) and recycled after eight reaction runs for degradation of MO (red) and RhB (blue), (b) TEM image of SGCN/Fe_3_O_4_/PVIs/Pd after eight cycles.
